# Complete genome sequences of two closely related isolates of *Staphylococcus saprophyticus* isolated from human fingertips

**DOI:** 10.1128/mra.00425-24

**Published:** 2024-06-25

**Authors:** S. Q. Zhang, K. M. Leung, G. K. K. Lai, S. D. J. Griffin

**Affiliations:** 1 Shuyuan Molecular Biology Laboratory, The Independent Schools Foundation Academy, Hong Kong, China; Department of Biological Sciences, Wellesley College, Wellesley, Massachusetts, USA

**Keywords:** *Staphylococcus saprophyticus*, *agrD*, IsaB

## Abstract

Complete genomes of two closely related isolates of *Staphylococcus saprophyticus* from human fingertips, SZ.YL11 and SZ.PL35w, were established through hybrid assembly. Each possesses a single circular chromosome and a circular plasmid, totaling 2,611,553 and 2,611,619 bp, respectively (with G + C 33.14% for both).

## ANNOUNCEMENT

The coagulase-negative staphylococcus, *Staphylococcus saprophyticus,* is a common Gram-positive commensal of the human skin microbiome but may also be uropathogenic, responsible for 10%–20% of uncomplicated urinary tract infections in young adult women (1). Two lineages, G and S, have been identified in the species, with a human origin proposed for the S-lineage (2).

Here, SZ.YL11 and SZ.PL35w were isolated from fingertips of different individuals in Hong Kong during a broad environmental survey of antibiotic-quaternary ammonium compound co-resistance (exempt from ethics committee review) (see Table 1). A moistened cotton bud used to swab under fingernails was subsequently rinsed with 1 mL 0.9% (wt/vol) saline, and 100 µL of this extract spread onto Luria agar (3) for overnight incubation. Resultant colonies were tested for growth on Luria agar containing 0.01% (wt/vol) benzalkonium chloride or 50 µg/mL tetracycline, and SZ.YL11 and SZ.PL35w, which showed resistance to both, were passaged to purity on Luria agar before a single colony of each was streaked onto this medium for overnight growth before harvesting for DNA extraction (DNeasy PowerSoil Pro Kit, Qiagen GmbH). All agar incubations were at 27°C (favoring environmentally mobile species).

**TABLE 1:** Samples, sequencing, and assembly data for SZ.YL11 and SZ.PL35w

*Samples*				
Isolate	SZ.YL11		SZ.PL35w	
Host	*Homo sapiens* (male)		*Homo sapiens* (female)	
Source	Fingertip swab		Fingertip swab	
Date of collection	2022-11-16		2022-11-22	
Location	22.2642601 N 114.1299767 E		22.2642601 N 114.1299767 E	

Sequencing				
Sequencing method	Illumina NovaSeq	Nanopore MinION	Illumina NovaSeq	Nanopore MinION
Raw reads	2,564,580 pairs	132,641	2,475,039 pairs	255,136
Mean length, bp	250	5,053	250	4,590
Total Mbp	1,282	670.3	1,238	1,171
*N*50	250	8,498	250	7,359
SRA (raw)	SRX23755333	SRX23755334	SRX23755463	SRX23755464
Filtered reads	2,563,321 pairs	78,931	2,473,940 pairs	156,220
Mean length	247.2	7,642.5	248.2	6,746.8
*N*50	250	9,385	250	8,122
Total Mbp	1,267	603.2	1,228	1,054

Assembly				
GenBank assembly	GCA_030845335.1		GCA_030845315.1	
Replicon	Chromosome SZ.YL11	Plasmid pSZ.YL11	Chromosome SZ.PL35w	Plasmid pSZ.PL35w
GenBank accession	CP132559	CP132560	CP132557	CP132558
Topology	Circular	Circular	Circular	Circular
Size	2,607,114	4,439	2,607,180	4,439
G + C %	33.14	30.07	33.14	30.07
Coverage	405×		231×	
Rotated to begin	*dnaA*	*not rotated*	*dnaA*	*not rotated*
CDs	2,497	3	2,497	3
Pseudogenes	68	0	67	0
rRNA (5S, 16S, 23S)	7, 6, 6	0	7, 6, 6	0
tRNA	60	0	60	0

Paired-end short-read sequencing libraries (NEBNext Ultra DNA Library Prep Kit, Illumina, Inc.) were sequenced via the NovaSeq 6000 platform using an SP PE250 flowcell and v1.5 Reagent Kit. Raw read pairs were quality filtered and trimmed using TrimGalore! v0.6.7 (stringency: 3; -e: 0.2) (https://github.com/FelixKrueger/TrimGalore). Long-read libraries, prepared from the same extracted DNA using the Rapid Barcoding Kit SQK-RBK004 (without size selection), were sequenced via Spot-ON Flow Cell (vR9), MinION sequencer, and MinKNOW v3.1.8 software, with basecalling by Guppy v2.1.3 high-accuracy mode (all by Oxford Nanopore Technologies plc). Final long-read data sets were trimmed by Filtlong v0.2.1 (https://github.com/rrwick/Filtlong) (min_length: 2,000; target_bases: 500 Mbp; length_weight: 10), selecting high-quality reads to scaffold the assembly. Default parameters were used for all software unless otherwise specified.

Hybrid assembly, overlap removal, and sequence rotation were performed by Unicycler v0.4.8-beta (4) yielding, for both isolates, a circular chromosome and a single circular plasmid that were submitted to NCBI PGAP v6.5 (5) for annotation. SZ.YL11 and SZ.PL35w share an average nucleotide identity of 99.70% [ANIb by BLAST+ 2.2.29 (6)] with *Staphylococcus saprophyticus* KS160 (SRX7665059), the reference strain for lineage-S (2). Sequencing and assembly data given in Table 1.

The 4,439-bp pT181-type *repC-mobV-tet(K*) plasmid (7) is identical in both isolates. However, the otherwise homologous SZ.YL11 and SZ.PL35w chromosomes were found by the Assembly Comparison Tool (8) to differ at two loci. SZ.PL35w carries an additional GAAATAGATTTT repeat in the accessory gene regulator gene *agrD*, where sequence variation is common and has been linked to pathogenicity (9). In addition, the immunodominant antigen B (IsaB_6) protein (10) encoded in SZ.PL35w contains an additional NMNQKDMSMNHNMDNMNG repeat, which is predicted by AlphaFold (11) to extend the stalk region of the surface protein (https://alphafold.ebi.ac.uk/entry/A0A380HP74), similar to the repetitive motifs of adhesin A in *Bartonella* (12). Chromosomal genes linked to biocide resistance, *norC*, *sdrM*, *sepA,* and *qacJ* (13, 14), were identified by RGI v6.0.3 in CARD (15) .

## Data Availability

Sequence data for *Staphylococcus saprophyticus* strains SZ.YL11 and SZ.PL35w are available through NCBI with relevant accession numbers given in Table 1.
